# When growth becomes risky: stress-driven sporulation and sexual development in fungi

**DOI:** 10.1007/s44154-026-00333-1

**Published:** 2026-08-04

**Authors:** Jie Yang, Qinhu Wang, Huiquan Liu

**Affiliations:** State Key Laboratory for Crop Stress Resistance and High-Efficiency Production, College of Plant Protection, Northwest A&F University, Yangling, Shaanxi 712100 China

**Keywords:** Fungal development, Sporulation, Sexual reproduction, Nutrient limitation, Stress signaling, RNA editing, Disease management

## Abstract

Fungi frequently encounter nutrient limitation, osmotic stress, oxidative pressure, light fluctuations, temperature shifts, and host-associated stresses. Rather than responding passively to deteriorating environments, many fungi actively redirect development from vegetative growth toward asexual sporulation, sexual reproduction, or resting structure formation. These transitions promote dispersal, dormancy, stress resistance, or genetic diversification, but they are not governed by a single conserved stress-response pathway. Instead, conserved nutrient- and stress-sensing modules are integrated with lineage-specific developmental circuits. This review synthesizes recent advances in understanding how environmental stress, particularly nutrient limitation, regulates fungal reproductive development. We discuss nutrient limitation as both a physiological constraint and a developmental signal, and compare regulatory mechanisms across yeasts, filamentous ascomycetes, and pathogenic fungi. Major pathways and regulatory layers include cAMP-PKA, TOR, HOG MAPK signaling, light-responsive systems, the Velvet complex, chromatin regulation, diffusible chemical signals, and sexual-stage-specific adenosine-to-inosine (A-to-I) mRNA editing. Together, these mechanisms determine whether fungi remain vegetative or commit to reproduction. We also evaluate why sexual reproduction may be favored under stressful or low-fitness conditions. While asexual spores and resting structures can enhance survival and dispersal, costly meiotic reproduction may be promoted when recombination allows offspring or alleles to escape maladapted genetic backgrounds. Fitness-associated sex and abandon-ship models therefore provide useful evolutionary frameworks for interpreting stress-induced reproduction. Understanding these mechanisms is timely and important because fungal reproductive switching affects industrial spore production, pathogen transmission, disease management, and evolutionary potential. Future progress will require causal, ecologically grounded models linking environmental perception, molecular regulation, reproductive output, and fitness consequences.

## Introduction

Fungal life histories are shaped by a recurring trade-off between local growth and future transmission. Vegetative growth allows fungi to exploit available resources, expand through substrates, and accumulate biomass. This strategy is effective only while local conditions continue to support further growth. When conditions deteriorate, the value of continued growth declines. Nutrients may become limiting, water availability may fall, oxidative damage may increase, or host tissues may impose immune-derived stress. Under such conditions, fungi may shift resources toward propagule formation, sexual reproduction, or dormancy (Adams et al. 1998; Park and Yu 2012; Neiman 2005). This shift is not a passive collapse of growth. In well-studied systems, it is controlled by defined developmental programs: conidiation in *Aspergillus nidulans* depends on the BrlA-AbaA-WetA cascade (Adams et al. 1998; Park and Yu 2012), rhythmic conidiation in *Neurospora crassa* is shaped by light and circadian regulation (Baker et al. 2012; Dunlap and Loros 2004), meiotic sporulation in *Saccharomyces cerevisiae* requires specific nutrient and mating-type conditions (Neiman 2005; Van Werven and Amon 2011; Honigberg and Purnapatre 2003), and sexual differentiation in *Schizosaccharomyces pombe* is induced by nitrogen starvation through Ste11-, Pat1-, and Mei2-dependent regulation (Mata et al. 2002; Otsubo and Yamamoto 2012; Yamamoto 1996, 2010).

**Fig. 1:**
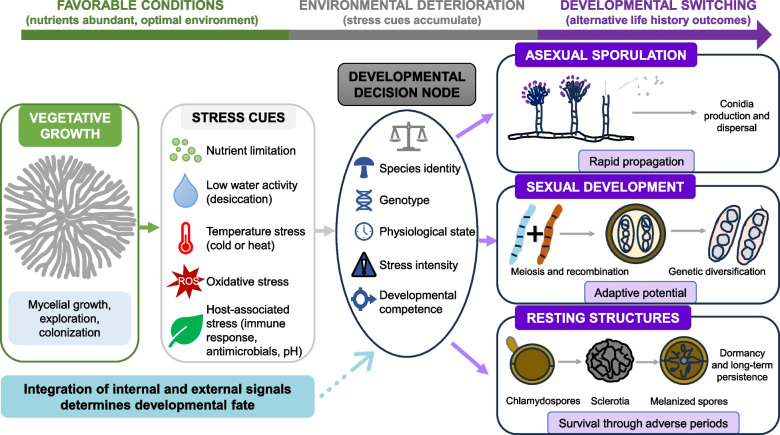
Environmental stress redirects fungal life-history decisions from vegetative growth to reproductive or persistent states. Under favorable conditions, fungi prioritize vegetative growth and local resource exploitation. When nutrients, water activity, temperature, oxidative conditions, or host-associated environments deteriorate, continued growth may become inefficient or risky. Depending on species identity, genotype, physiological state, stress intensity, and developmental competence, fungi may shift toward asexual sporulation, sexual development, or formation of stress-resistant resting structures. These developmental outputs promote dispersal, dormancy, persistence, or genetic diversification

In these scenarios, nutrient limitation operates in two interconnected modes: as a metabolic stress condition that passively restricts vegetative growth, and as an instructive developmental signal that actively drives genetic reprogramming (Honigberg and Purnapatre 2003; Loewith and Hall 2011). The selective pressure imposed by the physiological toll of the stress condition drives the evolution of precise sensing pathways, effectively transforming an environmental deficit into an anticipatory cue for fitness realignment. This adaptive duality underpins stress-driven developmental switching, which, as used here, refers to transitions from vegetative growth to asexual sporulation, sexual development, or stress-resistant resting structures. These outputs share a broad function: they help fungi persist beyond the current environment. However, they differ in mechanism and evolutionary consequence. Asexual spores generally preserve the parental genotype and support dispersal or survival (Dijksterhuis 2019). Resting structures such as chlamydospores and sclerotia support persistence through unfavorable periods (Willetts 1971). Sexual spores or meiotic products generate new genetic combinations through recombination and segregation (Mata et al. 2002; Neiman 2005; Otsubo and Yamamoto 2012; Yamamoto 1996, 2010). This distinction is important because overlapping environmental cues can lead to outcomes with very different genetic consequences (Fig. 1).

A central issue is how fungi convert environmental deterioration into developmental commitment. Several regulatory modules recur across fungal systems. cAMP-PKA and TOR pathways promote growth under nutrient-rich conditions and often repress starvation-associated development (Honigberg and Purnapatre 2003; Loewith and Hall 2011; Otsubo and Yamamoto 2012; Van Werven and Amon 2011; Yamamoto 1996). HOG MAPK pathways connect osmotic, oxidative, and nutritional stress to stress adaptation and developmental timing (Garrido-Bazán et al. 2018; Hohmann 2002; Lara‐Rojas et al. 2011; Mata et al. 2002; Otsubo and Yamamoto 2012; Saito 2004). Light-responsive systems and the Velvet complex coordinate development and secondary metabolism in aspergilli and other filamentous ascomycetes (Bayram et al. 2008; Bayram and Braus 2012; Calvo 2008; Kato et al. 2003). Chromatin regulators further influence developmental competence by controlling access to developmental and secondary-metabolite loci (Collemare and Seidl 2019; Gacek and Strauss 2012; Pfannenstiel and Keller 2019; Strauss and Reyes-Dominguez 2011).

In addition to chromatin-level and transcriptional regulation, recent studies have revealed a post-transcriptional layer of developmental control in filamentous ascomycetes. In *Sordariomycetes*, sexual-stage-specific adenosine-to-inosine (A-to-I) mRNA editing, mediated by an ADAR-independent Tad2-Tad3-Ame1 system, extensively recodes transcripts during fruiting-body development and ascosporogenesis (Liu et al. 2016; Feng et al. 2024). This mechanism does not simply add molecular detail to known developmental pathways; it provides a way to express reproduction-beneficial protein isoforms only during sexual development while avoiding their costs during vegetative growth. Thus, RNA editing offers a mechanistic bridge between developmental switching and the evolutionary problem of survival-reproduction trade-offs.

The evolutionary question is equally important: why reproduce, especially sexually, when conditions are poor? Asexual sporulation is often explained by resistance and dispersal. Sexual reproduction is harder to explain because it involves mating, meiosis, recombination, developmental time, and reduced direct transmission of parental allele combinations (Hadany and Beker 2003; Hadany and Otto 2009; Schoustra et al. 2010). Fitness-associated sex theory proposes that low-fitness individuals may benefit from recombination because sex allows alleles to escape maladapted genetic backgrounds (Hadany and Beker 2003; Hadany and Otto 2009; Schoustra et al. 2010). Experimental studies in *A. nidulans* support this idea by linking low fitness or fungicide stress with increased sexual reproduction or outcrossing (Schoustra et al. 2010; Seudre et al. 2018). The abandon-ship framework extends this logic by treating dispersal, dormancy, and sex as alternative escape routes from deteriorating conditions (Gerber and Kokko 2018; Seudre et al. 2018).

This review therefore focuses on four questions: how nutrient limitation triggers sporulation or sexual development; how stress and nutrient pathways integrate with developmental regulators; why sex may be favored under stressful or low-fitness conditions; and how these mechanisms can be used to improve fungal biotechnology and disease control.

## Nutrient limitation as a developmental signal

**Fig. 2:**
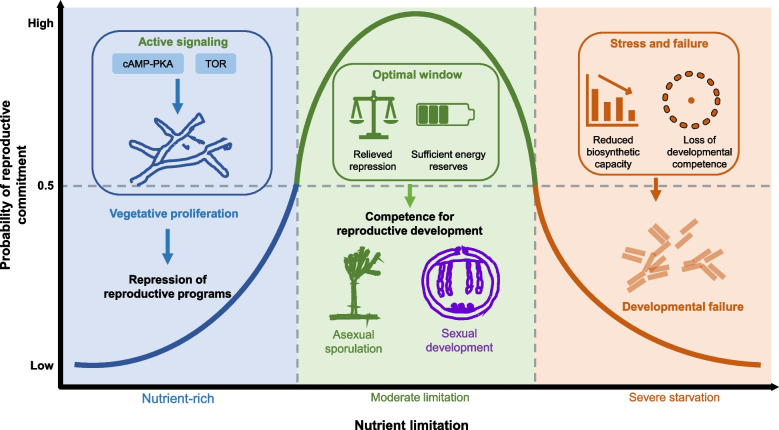
Nutrient limitation creates a developmental window for fungal sporulation and sexual development. Nutrient limitation acts both as a physiological challenge and as developmental information. Under nutrient-rich conditions, growth-promoting pathways such as cAMP-PKA and TOR favor vegetative proliferation. Moderate nutrient limitation can relieve repression of reproductive programs while leaving sufficient energy and biosynthetic capacity for sporulation or sexual development. Severe starvation, however, may reduce developmental competence and prevent completion of reproductive programs

Nutrient limitation is one of the most common contexts associated with fungal sporulation and sexual development. It should not be treated only as cellular damage. In many fungi, nutrient limitation also functions as information: it indicates that local growth is becoming less profitable and that dispersal, dormancy, or recombination may provide higher future returns. For instance, glucose depletion alone can bypass the white-opaque phenotypic switch in *Candida albicans*, directly linking a nutrient cue to a developmental fate decision (Guan et al. 2023). Nutrient limitation does not automatically trigger reproduction; however, severe starvation can prevent development because sporulation and sexual reproduction themselves require energy, biosynthetic precursors, and coordinated gene expression. The effect of nutrient limitation therefore depends on physiological competence: growth must be sufficiently unfavorable to justify switching, but cells must retain enough resources to complete the reproductive program (Honigberg and Purnapatre 2003; Neiman 2005; Van Werven and Amon 2011) (Fig. 2).

### Nutritional control of meiotic sporulation in budding yeast

The clearest example of nutrient-regulated sexual development is meiotic sporulation in *S. cerevisiae*. Cells usually enter meiosis only when they are diploid *MATa*/*MATα*, nitrogen-starved, depleted of fermentable carbon sources such as glucose, and supplied with a non-fermentable carbon source such as acetate (Honigberg and Purnapatre 2003; Neiman 2005; Van Werven and Amon 2011).

This combination is physiologically coherent. Nitrogen limitation reduces the value of mitotic proliferation. Glucose depletion relieves repression of respiratory and meiotic programs. Acetate provides energy for meiosis and spore-wall formation (Neiman 2005; Van Werven and Amon 2011). Sporulation is therefore not a nonspecific starvation response but a conditional developmental program.

cAMP-PKA and TOR signaling regulate this transition. Under nutrient-rich conditions, these pathways promote growth and repress stress-response and meiotic programs (Honigberg and Purnapatre 2003; Loewith and Hall 2011; Van Werven and Amon 2011). When nutrient conditions become unfavorable for mitotic proliferation, repression is relieved and meiotic regulators such as *IME1* and *IME2* are induced (Honigberg and Purnapatre 2003; Neiman 2005; Van Werven and Amon 2011). *IME1* activates meiotic transcription, whereas *IME2* promotes meiotic progression.

This system illustrates a broader principle: cells interpret nutrient signals combinatorially. Carbon quality, nitrogen availability, respiratory capacity, mating-type identity, and internal reserves jointly determine whether meiosis proceeds.

### Nitrogen starvation and sexual differentiation in fission yeast

Fission yeast provides a second major yeast model for nutrient-regulated reproductive switching. In *S. pombe*, nitrogen starvation is the principal cue that induces sexual differentiation. Under nutrient-rich conditions, cells grow mitotically. When nitrogen becomes limiting, compatible mating types can arrest in G1, mate, undergo karyogamy, enter meiosis, and produce spores (Mata et al. 2002; Otsubo and Yamamoto 2012; Yamamoto 1996, 2010).

The regulatory architecture differs from budding yeast. Nutrient sufficiency maintains growth-promoting cAMP-PKA and TOR signaling. Nitrogen starvation reduces these inputs and permits activation of Ste11, a high-mobility-group transcription factor that induces genes required for mating and meiosis (Mata et al. 2002; Otsubo and Yamamoto 2012; Yamamoto 1996, 2010). Meiotic entry is then controlled by the Pat1 kinase-Mei2 regulatory switch. During vegetative growth, Pat1 phosphorylates Mei2, an RNA-binding protein required for meiosis, targeting it for degradation. Upon nitrogen starvation and successful conjugation, the Pat1 inhibitor Mei3 is expressed, leading to Pat1 inactivation, Mei2 de-repression, and irreversible commitment to meiosis (Mata et al. 2002; Otsubo and Yamamoto 2012; Yamamoto 2010).

The comparison between *S. cerevisiae* and *S. pombe* is useful because both link nutrient limitation to sexual development, but they use different downstream regulators. Budding yeast relies on *IME1*/*IME2*-centered control, whereas fission yeast relies on Ste11, Pat1, Mei2 and Mei3. This supports a modular model: conserved nutrient-sensing pathways feed into species-specific reproductive circuits.

### Nutrient limitation and conidiation in filamentous ascomycetes

In filamentous fungi, nutrient limitation can favor asexual sporulation because further vegetative expansion becomes less profitable. However, nutrient state alone rarely explains developmental commitment. Spatial context, aeration, surface exposure, light, colony age, and developmental competence often determine whether a colony continues growth or forms spores.

*A. nidulans* remains the best-defined genetic model. Asexual development requires BrlA, AbaA, and WetA. BrlA initiates conidiophore development, AbaA regulates phialide differentiation, and WetA is required for spore maturation and long-term conidial viability (Adams et al. 1998; Park and Yu 2012). Upstream regulators such as FluG and Flb proteins connect colony state to activation of this central cascade (Adams et al. 1998).

Other filamentous ascomycetes broaden the picture. In *Sordaria macrospora*, a homothallic model for fruiting-body development, mutations affecting light response, autophagy, and developmental signaling impair protoperithecium or perithecium formation (Engh et al. 2010; Pöggeler et al. 2018). In *Podospora anserina*, nutrient status, autophagy, mitochondrial function, aging, and sexual development are closely linked (Silar 2013).

These model systems illustrate that stress- or nutrient-associated reproductive switching is mediated by diverse regulators and structures. Conidiophores, perithecia, protoperithecia, ascospores, and resting structures cannot be treated as interchangeable developmental outputs, even when triggered by similar environmental cues.

### Nutrient limitation in plant-pathogenic fungi

In plant pathogens, nutrient limitation often occurs together with host-derived stress. Pathogens encounter immune responses, reactive oxygen species, antimicrobial compounds, tissue senescence, and changing nutrient availability. These conditions can influence both infection-related development and spore production.

In *Magnaporthe oryzae*, conidia are central to rice blast disease spread, and infection-related development depends on surface sensing, appressorium formation, autophagy, and nutrient-related differentiation (Talbot 2003; Veneault-Fourrey et al. 2006). In *F. graminearum*, sexual development produces perithecia and ascospores regulated by light, nutrient conditions, and developmental signals. Perithecia on crop residues release airborne ascospores that initiate infections under favorable environmental conditions (Osborne and Stein 2007; Trail 2009). In necrotrophic pathogens such as *Botrytis cinerea*, conidiation is commonly associated with damaged or senescent host tissue, although humidity, light, surface conditions, and host context also shape sporulation (Schumacher 2016).

These examples show why pathogen sporulation should not be reduced to direct starvation sensing. In disease cycles, nutrient state interacts with host ecology, residue availability, microclimate, and developmental regulation. Understanding stress-driven sporulation in pathogens therefore requires connecting molecular development with epidemiology.

### Nutrient-regulated developmental switching in human fungal pathogens

Human fungal pathogens provide additional examples of nutrient-regulated developmental switching with direct clinical relevance. In *C. albicans*, the white-opaque epigenetic switch controls mating competence, biofilm formation, and host niche adaptation. Phosphate limitation activates the PHO pathway, which can induce the opaque state even in otherwise mating-incompetent a/α cells (Zheng et al. 2020). More strikingly, glucose depletion can bypass the white-opaque switch entirely; white cells become mating-competent without transitioning to opaque, challenging the long-held view that the switch is an obligate prerequisite for sex in this species (Miller and Johnson 2002; Guan et al. 2023). This directly illustrates the principle that nutrient limitation can function as information that reprograms developmental competence, rather than merely acting as cellular damage.

In *Cryptococcus neoformans*, sexual reproduction produces basidiospores that serve as the primary infectious propagules. Autophagy is essential for this process: all 14 core *ATG* genes are required for meiotic progression and basidiospore production during bisexual mating (Jiang et al. 2020). The HAP (heme activator protein) complex links iron homeostasis, a form of nutrient stress, to sexual development by directly repressing the pheromone-responsive Cpk1 MAPK pathway (Kim and Bahn 2022). These connections between nutrient sensing, stress signaling, and sexual development parallel the circuits described in yeast and filamentous models, but they operate under host-imposed selection pressures that add a layer of complexity absent from free-living systems.

## Molecular integration of stress and development

Stress-driven reproductive development is controlled by interacting regulatory modules: Nutrient-sensing pathways influence whether cells remain growth-oriented; stress-activated pathways protect cells and modulate developmental timing; light and circadian systems align reproduction with environmental opportunity; the Velvet complex coordinates development with secondary metabolism; chromatin regulation shapes competence by controlling access to developmental genes (Table 1).

**Table 1:** Fungal developmental regulators and reproductive outputs surveyed in this review

Species	Nutrient signal	Core developmental regulators	Developmental outputs	References
*Saccharomyces cerevisiae*	Nitrogen limitation; non-fermentable carbon (acetate)	IME1, IME2; cAMP-PKA; TOR	Meiotic ascospores	Honigberg and Purnapatre 2003; Neiman 2005; Van Werven and Amon 2011
*Schizosaccharomyces pombe*	Nitrogen starvation	Ste11, Pat1, Mei2, Mei3; cAMP-PKA; TORC1/TORC2	Mating, karyogamy, meiosis, spores	Mata et al. 2002; Otsubo and Yamamoto 2012; Yamamoto 1996, 2010
*Aspergillus nidulans*	Carbon/nitrogen balance; light	BrlA, AbaA, WetA; FluG, Flb; VeA, VelB, LaeA; SakA/HogA, MpkC	Conidia (asexual); ascospores (sexual)	Adams et al. 1998; Park and Yu 2012; Bayram et al. 2008; Garrido-Bazán et al. 2018
*Neurospora crassa*	Light; circadian rhythm	WC-1, WC-2 (White Collar Complex); FRQ	Rhythmic conidia	Baker et al. 2012; Dunlap and Loros 2004
*Sordaria macrospora*	Light; autophagy	Fruiting-body developmental regulators	Protoperithecia, perithecia, ascospores	Engh et al. 2010; Pöggeler et al. 2018
*Podospora anserina*	Nutrient status; autophagy; mitochondrial function	Aging-associated developmental regulators	Sexual development	Silar 2013
*Fusarium graminearum*	Light; nutrient conditions	White Collar Complex; Velvet-family regulators; MAPK pathways; Tad2, Tad3, Ame1 (A-to-I mRNA editing)	Perithecia, ascospores (sexual); conidia (asexual)	Osborne and Stein 2007; Trail 2009; Kim et al. 2015; Jiang et al. 2012; Liu et al. 2016; Feng et al. 2024
*Magnaporthe oryzae*	Surface sensing; autophagy; nutrient status	cAMP; MAPK pathways	Conidia; appressoria	Talbot 2003; Veneault-Fourrey et al. 2006; Lengeler et al. 2000
*Botrytis cinerea*	Host tissue senescence; humidity; light	MAPK cascades	Conidia; sclerotia	Schumacher 2016
*Candida albicans*	Phosphate limitation; glucose depletion	PHO pathway; white-opaque epigenetic switch	White-opaque switching; sexual mating	Zheng et al. 2020; Guan et al. 2023
*Cryptococcus neoformans*	Iron homeostasis	14 core ATG genes; HAP complex; Cpk1 MAPK	Basidiospores (sexual)	Jiang et al. 2020; Kim and Bahn 2022

### cAMP-PKA signaling

cAMP-PKA signaling generally promotes growth when nutrients are favorable, but its developmental output depends on species and cell type.

In *S. cerevisiae*, cAMP-PKA signaling promotes growth and suppresses stress-response and meiotic programs under nutrient-rich conditions (Honigberg and Purnapatre 2003; Neiman 2005; Van Werven and Amon 2011). Reduced PKA activity under starvation contributes to meiotic competence. In *S. pombe*, cAMP-PKA signaling also represses sexual differentiation when nutrients are abundant; nitrogen starvation permits Ste11-dependent induction of mating and meiotic genes (Mata et al. 2002; Otsubo and Yamamoto 2012; Yamamoto 1996, 2010).

In filamentous and pathogenic fungi, cAMP-PKA signaling has broader developmental roles. In *A. nidulans*, conidiation depends on interactions between nutrient state, environmental cues, and developmental regulators rather than cAMP-PKA alone (Adams et al. 1998; Park and Yu 2012). In *M. oryzae*, cAMP signaling promotes appressorium formation, showing that the same general signaling module can support infection-related morphogenesis rather than simply repress reproduction (Lengeler et al. 2000; Talbot 2003).

Thus, cAMP-PKA should be treated as a nutrient-sensitive regulatory module, not as a universal on/off switch for sporulation or sex.

### TOR signaling

TOR signaling promotes protein synthesis, ribosome biogenesis, metabolism, and cell growth when nutrients are available (Loewith and Hall 2011). In *S. cerevisiae*, TOR responds strongly to nitrogen and amino acid availability and helps maintain cells in a growth-committed state (Honigberg and Purnapatre 2003; Loewith and Hall 2011; Van Werven and Amon 2011). Reduced TOR signaling contributes to starvation responses, autophagy, and meiotic competence.

In *S. pombe*, the two TOR complexes have opposing roles in sexual development: TORC1 (containing Tor2) supports vegetative growth and represses sexual differentiation under nutrient-rich conditions, whereas TORC2 (containing Tor1) is required for proper G1 arrest and the onset of sexual development upon nitrogen starvation (Mata et al. 2002; Otsubo and Yamamoto 2012; Yamamoto 1996). This reinforces the view that TOR functions as a nutrient-state interpreter.

In filamentous fungi, TOR influences growth, metabolism, and development (Wang et al. 2023; Yu et al. 2014). Reduced TOR activity may permit developmental reprogramming when reserves remain sufficient, but excessive starvation can impair sporulation because spore formation itself requires energy and biosynthesis. TOR therefore regulates the balance between growth and developmental competence rather than directly specifying a single reproductive fate.

### HOG MAPK signaling

HOG MAPK pathways connect environmental stress to transcriptional adaptation, osmotic balance, stress tolerance, and developmental timing.

In *S. cerevisiae*, Hog1 mediates osmotic stress adaptation by regulating glycerol accumulation and stress-responsive transcription (Hohmann 2002; Saito 2004). In *S. pombe*, the Sty1/Spc1 stress-activated MAPK pathway regulates responses to osmotic, oxidative, and nutritional stress through transcription factors such as Atf1 (Honigberg and Purnapatre 2003; Mata et al. 2002). In *A. nidulans*, SakA/HogA interacts with AtfA to regulate stress-response genes, conidial stress resistance, germination, and development (Lara‐Rojas et al. 2011). The related MAPK MpkC has overlapping and distinct roles, and genetic studies show that SakA and MpkC can have shared or opposing functions during stress adaptation and development (Garrido-Bazán et al. 2018).

These examples show that HOG pathways are stress-integration modules. They do not universally trigger sporulation. Instead, they influence whether cells survive stress, delay germination, remain vegetative, or become developmentally competent, depending on fungal system and physiological state.

### Light and circadian regulation

Light provides information about spatial position and environmental timing. For surface-growing fungi, light exposure can indicate aerial conditions suitable for spore dispersal. Daily light cycles also correlate with humidity and temperature.

In *N. crassa*, the White Collar Complex, composed of WC-1 and WC-2, regulates blue-light responses and interacts with the FRQ-based circadian clock to generate rhythmic conidiation (Baker et al. 2012; Dunlap and Loros 2004). This system demonstrates how fungi can time reproduction according to predictable environmental cycles.

In *A. nidulans*, light generally favors asexual conidiation, whereas darkness often promotes sexual development (Bayram et al. 2008; Bayram and Braus 2012; Calvo 2008; Kato et al. 2003). This bias is mediated through photoreceptors and the Velvet system, but the outcome also depends on medium, oxygen, strain background, developmental stage, and nutrient state (Bayram et al. 2008; Bayram and Braus 2012; Calvo 2008). In *F. graminearum*, the White Collar Complex acts as a negative regulator of sexual development; deletion of its components can increase perithecium formation even under normally unfavorable conditions (Kim et al. 2015), and ascospores from perithecia are central to *Fusarium* head blight (FHB) epidemiology (Osborne and Stein 2007; Trail 2009). In *S. macrospora*, fruiting-body development is also shaped by light-responsive and developmental pathways (Engh et al. 2010; Pöggeler et al. 2018).

Light therefore acts as an ecological and developmental cue, but its effect is interpreted through species-specific regulatory networks.

### The Velvet complex

The Velvet complex is a fungal regulatory system linking light, development, and secondary metabolism. In *A. nidulans*, VeA, VelB, and LaeA form a nuclear complex that promotes sexual development and regulates secondary metabolite gene clusters (Bayram et al. 2008; Bayram and Braus 2012; Keller et al. 2005). VeA nuclear accumulation is favored in darkness, supporting sexual development; light reduces nuclear VeA accumulation and biases development toward asexual conidiation (Bayram et al. 2008; Bayram and Braus 2012; Calvo 2008; Kato et al. 2003).

Velvet regulation is important because fungal reproduction often coincides with production of pigments, toxins, antibiotics, or protective metabolites. In *A. nidulans*, veA influences sterigmatocystin and penicillin gene expression, directly linking morphology and secondary metabolism (Kato et al. 2003). Velvet-family regulators also affect development, secondary metabolism, and pathogenicity-related traits in other filamentous ascomycetes, although the regulatory wiring differs among species (Bayram and Braus 2012; Calvo 2008; Jiang et al. 2012).

The Velvet system illustrates a broader principle: reproductive outcome is rarely determined by one pathway. Nutrient state, light, stress signaling, developmental competence, and chromatin context jointly determine whether a colony produces asexual spores, sexual structures, or remains vegetative.

### Chromatin and transcriptional competence

Developmental switching requires large-scale transcriptional reprogramming. Genes for vegetative growth must be downregulated or reprioritized, while genes for conidiophore development, meiosis, spore-wall formation, stress resistance, or secondary metabolism must become accessible and active.

Chromatin regulation contributes to this process by controlling gene accessibility (Collemare and Seidl 2019; Gacek and Strauss 2012; Pfannenstiel and Keller 2019; Strauss and Reyes-Dominguez 2011). Histone acetylation is often associated with open chromatin and active transcription, whereas histone deacetylases, methyltransferases, and chromatin remodelers can repress, activate, or poise developmental and secondary-metabolite loci depending on context (Collemare and Seidl 2019; Gacek and Strauss 2012; Pfannenstiel and Keller 2019; Strauss and Reyes-Dominguez 2011). In *Aspergillus* and *Fusarium*, chromatin regulators influence secondary metabolism, pathogenicity, and developmental traits (Atanasoff-Kardjalieff and Studt 2022; Collemare and Seidl 2019; Gacek and Strauss 2012; Pfannenstiel and Keller 2019; Strauss and Reyes-Dominguez 2011). A comprehensive survey of chromatin and transcriptional control of fruiting-body development across *N. crassa*, *A. nidulans*, *S. macrospora*, and *S. commune* identified conserved chromatin-modifier families and transcription factor networks that distinguish vegetative from sexual developmental programs (Nowrousian 2022).

The most accurate view is not that one histone mark universally triggers reproduction. Rather, chromatin state creates or restricts developmental competence at specific loci. Stress and nutrient pathways likely act through transcription factors, kinase cascades, and chromatin regulators to determine whether reproductive genes can be activated.

### Post-transcriptional recoding by A-to-I mRNA editing

Developmental switching is not controlled only by signaling pathways, transcription factors, and chromatin state. In *Sordariomycetes*, sexual development is also accompanied by extensive post-transcriptional recoding through A-to-I mRNA editing (Wang et al. 2016; Liu et al. 2016, 2017; Bian et al. 2019). Because inosine is read as guanosine during translation, A-to-I editing can generate transcript-level A-to-G changes that alter codons without changing the genome.

This fungal editing system is distinct from ADAR-mediated A-to-I editing in animals. It is ADAR-independent, occurs mainly during sexual development, and is strongly enriched in coding sequences. Recent work showed that it is mediated by a Tad2-Tad3-Ame1 complex, in which the fungal-specific Ame1 cofactor enables the conserved tRNA-editing enzymes Tad2 and Tad3 to act on mRNA substrates (Feng et al. 2024). The emergence of Ame1 in the most recent common ancestor of *Sordariomycetes* suggests that this system may have evolved together with lineage-specific innovations in fruiting-body development and ascospore formation (Feng et al. 2024; Du et al. 2025).

Functionally, A-to-I editing provides a flexible way to tune the proteome during sexual reproduction. It can generate nonsynonymous changes, restore premature stop codons, and modulate protein abundance or function. Experimental studies in *Fusarium* and *Neurospora* show that editing contributes to perithecium development, meiosis, ascospore delimitation, and ascospore maturation, including through targets such as Dbf2, Mus81, and Spo11 (Liu et al. 2016, 2017; Xin et al. 2023; Qi et al. 2024; Du et al. 2026; Wu et al. 2026a, 2026b).

Although A-to-I editing does not function as an upstream environmental sensor, it directly interfaces with stress adaptation by acting as a sexual-stage-specific tuning gate. By restricting reproduction-beneficial protein variants or dosage modifications strictly to the reproductive phase, editing effectively neutralizes their potential fitness costs during vegetative growth. Crucially, this post-transcriptional partitioning allows fungi to safeguard robust vegetative stress resilience—such as Mus81-dependent heat tolerance or Spo11-mediated mitotic fitness—while concurrently fulfilling the distinct genetic requirements of meiosis (Wu et al. 2026a, 2026b). Consequently, fungal A-to-I mRNA editing provides an elegant, non-genomic strategy to circumvent antagonistic pleiotropy, offering a definitive mechanistic bridge between stress-driven developmental switching and the evolutionary resolution of survival-reproduction trade-offs (Xin et al. 2025).

### Diffusible signals and colony-level coordination

Beyond protein-centered and RNA-based regulatory modules, diffusible small molecules also contribute to developmental coordination. Fungal oxylipins and aromatic alcohols can act as hormone-like or quorum-sensing signals that translate colony density, age, metabolic state, and local microenvironment into reproductive commitment (Chen and Fink 2006; Niu et al. 2020; Tsitsigiannis and Keller 2007). Together with nutrient signaling, stress-activated pathways, chromatin regulation, and post-transcriptional RNA editing, these chemical signals form an integrated network through which environmental deterioration is converted into developmental output.

## Why sex can be favored under stress

Asexual sporulation under stress is relatively easy to explain: spores can survive or disperse better than vegetative cells (Dijksterhuis 2019; Wyatt et al. 2013). Sexual reproduction is harder to explain because it can impose substantial costs. These include mating, meiosis, recombination, developmental time, partner finding, and the breakdown of successful allele combinations. The classic two-fold cost of sex further emphasizes that sexual reproduction can reduce the short-term transmission advantage of a well-adapted genotype (Hadany and Beker 2003; Hadany and Otto 2009; Schoustra et al. 2010).

Molecular mechanisms that resolve survival-reproduction conflicts are especially important for evaluating these evolutionary explanations. In *Sordariomycetes*, sexual-stage-specific A-to-I mRNA editing provides one such mechanism. By allowing reproduction-beneficial protein forms to be produced only during sexual development, editing can reduce the vegetative costs of genes required for meiosis, ascus formation, or ascospore maturation (Feng et al. 2024; Qi et al. 2024; Du et al. 2026; Wu et al. 2026a, 2026b). Thus, the adaptive value of sex under stress may depend not only on recombination and spore resistance, but also on molecular systems that make costly reproductive programs conditionally deployable.

### Limits of resistance and dispersal explanations

Physical resistance and dispersal explain many reproductive transitions. *Aspergillus* conidia and yeast ascospores are more stress-tolerant than actively growing cells in many contexts (Dijksterhuis 2019; Wyatt et al. 2013). In *S. pombe*, nitrogen starvation-induced meiosis produces spores adapted for survival under nutrient limitation (Mata et al. 2002; Otsubo and Yamamoto 2012; Yamamoto 1996, 2010). Resting structures such as chlamydospores and sclerotia provide temporal persistence in many soilborne and plant-associated fungi (Willetts 1971).

However, resistance alone does not explain why costly sex is favored when clonal spores can also disperse or survive. In *S. cerevisiae*, meiotic ascospores have protective spore walls, so physical survival is clearly part of the benefit of sporulation (Neiman 2005). In *A. nidulans*, experimental evidence indicates that stress-associated sexual reproduction cannot be reduced to spore durability. Sexual reproduction is associated with low-fitness conditions (Schoustra et al. 2010), and sublethal fungicide stress can increase outcrossing, with recombinant offspring showing improved performance under stress (Seudre et al. 2018).

These findings support a more specific interpretation: in facultatively sexual fungi, stress-associated sex may be favored when recombination improves descendant genotypes, not merely when sexual spores are physically resistant.

### Fitness-associated sex

Fitness-associated sex theory proposes that low-fitness individuals may reproduce sexually because recombination allows alleles to escape maladapted genetic backgrounds (Hadany and Beker 2003; Hadany and Otto 2009; Schoustra et al. 2010). In a clonal lineage, beneficial and deleterious alleles remain linked. If the current genotype performs poorly under a new environment, clonal reproduction preserves that poor combination. Sex can break these associations and generate offspring with new allele combinations.

Experimental work in *A. nidulans* provides direct support. Sexual reproduction is associated with low-fitness conditions, and fungicide stress can increase outcrossing while producing recombinant offspring with improved performance under stress (Schoustra et al. 2010; Seudre et al. 2018). These studies are especially valuable because they connect reproductive mode, stress, recombination, and descendant performance.

Fission yeast offers a mechanistic example of condition-dependent sexual differentiation: nitrogen starvation induces mating and meiosis through defined pathways (Mata et al. 2002; Otsubo and Yamamoto 2012; Yamamoto 1996, 2010). However, demonstrating fitness-associated sex in the evolutionary sense requires additional measurements of genotype-specific fitness, recombination outcomes, and offspring performance under the inducing stress.

The key point is that the benefit of sex may not appear as improved survival of the parent. The beneficiaries may be recombinant offspring or alleles that escape a low-fitness genetic background (Hadany and Beker 2003; Hadany and Otto 2009; Schoustra et al. 2010; Seudre et al. 2018).

### Abandon-ship and escape strategies

**Fig. 3:**
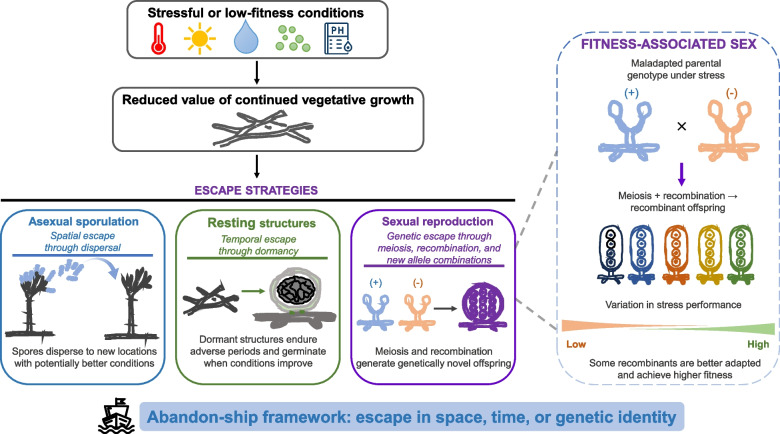
Evolutionary explanations for stress-associated sexual reproduction in fungi. Stressful or low-fitness conditions can reduce the value of continued vegetative growth and favor alternative escape strategies. Asexual spores provide spatial escape through dispersal, whereas resting structures provide temporal escape through dormancy. Sexual reproduction provides genetic escape by recombination and segregation, allowing alleles or offspring to escape maladapted genetic backgrounds. Fitness-associated sex and abandon-ship models offer complementary explanations for why facultatively sexual fungi may increase sexual reproduction or outcrossing under stressful conditions

The abandon-ship framework proposes that organisms in poor condition may increase investment in escape routes rather than continue investing in a declining state (Gerber and Kokko 2018; Seudre et al. 2018). These routes include escape in space through dispersal, escape in time through dormancy, and escape in genetic identity through sex (Gerber and Kokko 2018) (Fig. 3).

Fungi fit this framework conceptually: Conidia provide spatial escape; resting structures provide temporal escape; sexual reproduction provides genetic escape through recombination. Direct experimental tests remain limited but suggestive: in *A. nidulans*, stress-associated outcrossing supports the genetic-escape component of this framework, though broader testing across additional species and stress regimes is needed (Gerber and Kokko 2018; Schoustra et al. 2010; Seudre et al. 2018). In *S. pombe*, nitrogen starvation-induced mating and sporulation provide a physiological example of shifting from proliferation to stress-resistant sexual spores (Mata et al. 2002; Otsubo and Yamamoto 2012; Yamamoto 1996, 2010), although strict abandon-ship interpretation requires direct fitness tests.

This framework should not be treated as a molecular pathway. It is an evolutionary interpretation. Testing it requires measuring whether low-condition individuals increase escape investment and whether the resulting propagules or offspring perform better than continued parental growth (Gerber and Kokko 2018; Seudre et al. 2018).

### What should be measured?

Distinguishing among adaptive explanations requires more than counting spores. Experiments should identify the reproductive mode: asexual sporulation, selfing, outcrossing, resting-structure formation, or mixed outcomes. This distinction is essential because these modes have different genetic consequences.

Propagule quality should also be measured. Viability, stress resistance, germination timing, dormancy depth, and dispersal capacity determine whether increased sporulation improves persistence (Dijksterhuis 2019; Wyatt et al. 2013). For sexual development, offspring performance is critical. Recombinant offspring should be tested under the inducing stress and alternative environments. In *A. nidulans*, this approach showed that increased outcrossing under fungicide stress is meaningful because recombinant progeny can perform better under stress (Seudre et al. 2018).

Parental condition must also be quantified. Growth rate, nutrient reserves, oxidative damage, survival probability, and stress tolerance help distinguish adaptive switching from physiological collapse. Without these measurements, stress-induced reproduction can be misinterpreted.

## Applied implications

Understanding stress-driven sporulation and sexual development has practical value for fungal biotechnology and disease management. The strongest applications are species-specific: they use known developmental biology rather than assuming a universal stress response.

### Optimizing industrial spore production

Industrial fungal processes often require high yields of viable and stress-tolerant propagules. These include biological control fungi, agricultural inoculants, fermentation starters, and products where spores are the commercial unit. The central challenge is to balance biomass accumulation with sporulation induction.

A rational strategy separates production into two phases. The first phase promotes vegetative biomass through favorable nutrients, aeration, moisture, pH, and temperature. The second phase applies controlled developmental cues that induce sporulation without causing severe damage. In aspergilli, surface exposure, aeration, light, nutrient state, and developmental competence influence conidiation (Adams et al. 1998; Bayram et al. 2008; Bayram and Braus 2012; Calvo 2008; Park and Yu 2012). In biocontrol and entomopathogenic fungi such as *Trichoderma*, *Metarhizium*, and *Beauveria*, carbon/nitrogen balance, water activity, aeration, surface culture, and light can affect conidial yield and quality (Gao and Liu 2010).

Mechanistic markers could improve production timing. Expression of conidiation regulators, nutrient-response genes, stress-response genes, or Velvet-associated regulators may indicate when cultures are ready to shift from growth to sporulation. Because regulatory outputs vary among species, conditions optimized for *A. nidulans* should not be assumed to maximize spore yield in *Metarhizium*, *Trichoderma*, or *Beauveria*.

### Reducing pathogen sporulation and disease spread

Many fungal diseases spread through repeated cycles of spore production. In *M. oryzae*, conidia are central to rice blast dissemination, and infection-related development depends on surface sensing, appressorium formation, autophagy, and host colonization (Talbot 2003; Veneault-Fourrey et al. 2006). In *F. graminearum*, perithecia and ascospores produced on crop residues contribute to FHB epidemics (Osborne and Stein 2007; Trail 2009). Reducing ascospore production can therefore reduce inoculum and slow disease spread.

Anti-sporulation strategies span a spectrum of specificity. The most downstream, single-purpose molecular interventions focus on development-specific transcription factors such as BrlA and WetA in aspergilli, which affect conidiation without impairing vegetative growth (Adams et al. 1998; Park and Yu 2012), though such regulators vary considerably across pathogen taxa. Signaling pathway nodes such as MAPK cascades and Velvet-associated regulators offer broader-spectrum targets but carry greater risk of off-target effects in beneficial fungi (Bayram et al. 2008; Bayram and Braus 2012; Garrido-Bazán et al. 2018; Lara‐Rojas et al. 2011). At the broadest level, environmental management, including light spectrum, photoperiod, humidity, leaf wetness, airflow, and residue management, can influence reproductive windows without imposing direct molecular selection pressure, though efficacy depends on field conditions (Kim et al. 2015; Osborne and Stein 2007; Trail 2009).

However, deploying these anti-sporulation interventions across the spectrum involves distinct ecological and evolutionary challenges. Because many master developmental regulators and signaling networks are evolutionarily conserved, molecular interventions carry a persistent risk of disrupting beneficial fungi within the agroecosystem. Furthermore, under highly variable field conditions, the prolonged application of targeted anti-sporulation agents exerts strong selective pressure, which can drive rapid pathogen adaptation. This evolutionary escape typically manifests as altered sporulation kinetics, the activation of cryptic alternative reproductive pathways, or a compensatory shift toward less susceptible life-cycle stages. Consequently, to ensure control durability and mitigate non-target risks, these approaches must be rigorously evaluated under realistic ecological conditions and integrated within broader, multi-tactical disease management frameworks.

## Future directions

While current research has delineated the broad molecular pathways and evolutionary frameworks governing stress-associated fungal reproduction, future work must transition from descriptive paradigms toward causal, predictive, and ecologically grounded models of reproductive commitment. Building these integrative models requires moving beyond static, bulk analyses to pair multi-dimensional environmental gradients—systematically varying nutrients, physical stresses, and population parameters—with high-resolution temporal tracking. By harnessing time-resolved multi-omics, targeted functional perturbations, and predictive machine learning architectures, the field can begin to decipher the precise, non-linear rules dictating cell-fate trajectories and life-history trade-offs. To bridge the gap between descriptive mechanization and actionable manipulation, future research should focus on several interconnected priorities spanning molecular, spatiotemporal, epitranscriptomic, and evolutionary scales.

First, the field needs to identify the molecular nodes that convert stress perception into irreversible developmental commitment. Many nutrient- and stress-responsive pathways have been implicated in fungal reproduction, including cAMP-PKA, TOR, HOG MAPK, light-responsive systems, the Velvet complex, and species-specific developmental regulators. However, in many fungi the direct links between environmental sensing and commitment remain poorly defined. Key questions are which transcription factors act immediately downstream of stress signaling, which kinase substrates control the growth-to-reproduction transition, which chromatin regulators establish developmental competence, and which genes distinguish stress-induced reproduction from general stress tolerance. Addressing these questions will require perturbation-based approaches rather than expression profiling alone.

Second, reproductive switching should be studied as a dynamic and spatially heterogeneous process. Stress intensity, duration, timing, and order can produce different developmental outcomes, even when they activate overlapping signaling pathways. In filamentous fungi, colonies also contain physiologically distinct regions, including peripheral hyphae, aging centers, aerial hyphae, conidiophores, sexual initials, fruiting bodies, and dormant structures. Bulk measurements can therefore obscure the small cell populations that first commit to reproduction. Time-resolved transcriptomics, phosphoproteomics, metabolomics, chromatin profiling, live imaging, and single-cell or spatially resolved approaches will be essential for distinguishing reversible stress responses from developmental commitment. Recent single-cell and pre-dormancy transcriptional studies show that such approaches are increasingly feasible in fungal systems (Tsuyuzaki et al. 2020; Wang et al. 2021).

Third, future models should incorporate post-transcriptional regulation, especially sexual-stage-specific A-to-I mRNA editing in *Sordariomycetes*. The Tad2-Tad3-Ame1 editing system provides a mechanism by which transcripts can be recoded specifically during sexual development, allowing fungi to tune protein function, restore reproductive proteins, or adjust protein dosage without changing the genome. A major open question is how editing activity is connected to upstream developmental signals such as nutrient state, stress perception, fruiting-body formation, meiosis, and ascospore differentiation. Comparative studies across *Fusarium, Neurospora, Sordaria, Podospora*, and related taxa could clarify whether A-to-I editing evolved primarily with complex sexual development, with the resolution of survival-reproduction trade-offs, or with both processes.

Fourth, evolutionary hypotheses about stress-associated sex require direct fitness tests. Fitness-associated sex and abandon-ship models predict that organisms in poor condition may increase investment in sex, dispersal, or dormancy, and that the resulting offspring or propagules should perform better than continued parental growth under relevant conditions (Gerber and Kokko 2018; Hadany and Beker 2003; Hadany and Otto 2009; Schoustra et al. 2010; Seudre et al. 2018). Testing these ideas requires more than measuring spore number. Future experiments should distinguish asexual sporulation, selfing, outcrossing, resting-structure formation, and mixed strategies; quantify parental condition; and measure offspring or propagule performance under both inducing and non-inducing environments. Similar tests across *Fusarium*, *Sordaria*, fission yeast, and other facultatively sexual fungi would reveal how broadly stress-associated sex improves descendant fitness.

Finally, mechanistic studies should be connected to practical outcomes in disease control and fungal biotechnology. In plant and human pathogens, small changes in sporulation timing, sexual reproduction, or propagule quality can strongly influence transmission and epidemic dynamics. In industrial systems, developmental markers could improve the timing of transitions from biomass accumulation to propagule production. The most useful applied models will therefore link environmental cues, molecular switches, reproductive output, propagule quality, and ecological performance. Such integration would allow fungal development to be predicted and manipulated rather than only described.

## Conclusion

Fungal sporulation and sexual development are regulated life-history decisions rather than passive consequences of environmental deterioration. When local growth becomes less profitable because of nutrient limitation, stress, host-derived pressure, or colony aging, fungi can redirect resources toward dispersal, dormancy, or sexual reproduction. These alternatives differ in their immediate costs and long-term evolutionary consequences, but they share a common logic: they allow fungi to persist beyond declining local conditions.

At the molecular level, reproductive switching emerges from the integration of multiple regulatory layers. Nutrient-sensitive pathways such as cAMP-PKA and TOR, stress-activated MAPK cascades, light and circadian systems, the Velvet complex, chromatin regulators, diffusible chemical signals, and post-transcriptional mechanisms together determine whether cells remain vegetative or become developmentally committed. Recent work on *Sordariomycetes* A-to-I mRNA editing adds an important layer to this framework by showing that sexual development can involve not only changes in gene expression, but also stage-specific reinterpretation of transcripts. Through protein recoding, stop-codon restoration, and dosage tuning, RNA editing can help resolve antagonistic pleiotropy between vegetative survival and sexual reproduction.

At the evolutionary level, stress-associated reproduction should be interpreted in terms of both parental escape and descendant performance. Asexual spores and resting structures can provide resistance, dispersal, and dormancy, whereas sexual reproduction can generate recombinant offspring that escape maladapted genetic backgrounds. Fitness-associated sex and abandon-ship models therefore complement mechanistic studies by explaining why costly reproductive programs may be favored under poor conditions. The key challenge is to connect molecular switching mechanisms with measurable fitness outcomes across environments and generations.

The practical implications are substantial. In biotechnology, understanding developmental commitment can improve the controlled production of viable, stress-tolerant propagules. In agriculture and medicine, disrupting pathogen sporulation or sexual reproduction can reduce inoculum, transmission, and evolutionary potential. Future progress will require causal gene-level models that connect environmental perception, signaling networks, chromatin state, RNA-level regulation, reproductive development, and ecological fitness. Such integrated models will move the field from describing stress-associated reproduction to predicting and manipulating fungal life-history decisions.

## Data Availability

Not applicable.

## Abbreviations

cAMP: Cyclic Adenosine Monophosphate
PKA: Protein Kinase A
TOR: Target of Rapamycin
TORC1/2: TOR Complex 1/2
MAPK: Mitogen-Activated Protein Kinase
HOG: High Osmolarity Glycerol
ADAR: Adenosine Deaminase Acting on RNA
A-to-I: Adenosine-to-Inosine
ATG: Autophagy-related
MAT: Mating Type
FRQ: Frequency
WC-1/2: White Collar-1/2
HAP: Heme Activator Protein
PHO: Phosphate
IME1/2: Inducer of Meiosis 1/2
FHB: *Fusarium* Head blight
